# Advancing Global Oncology Readership Needs: Pushing Forward Since 2015

**DOI:** 10.1200/GO.22.00414

**Published:** 2023-02-13

**Authors:** Melinda Mushonga, Hongcheng Zhu, Mary Gospodarowicz, Gilberto Lopes

**Affiliations:** ^1^ Sunnybrook Health Sciences, Odette Cancer Centre, Toronto, Canada; ^2^ Fudan University Shanghai Cancer Center, Shanghai, China; ^3^ Princess Margaret, Toronto, Canada; ^4^ Sylvester Comprehensive Cancer Center at the University of Miami, Miami, FL

## Introduction

**FIG 1:**
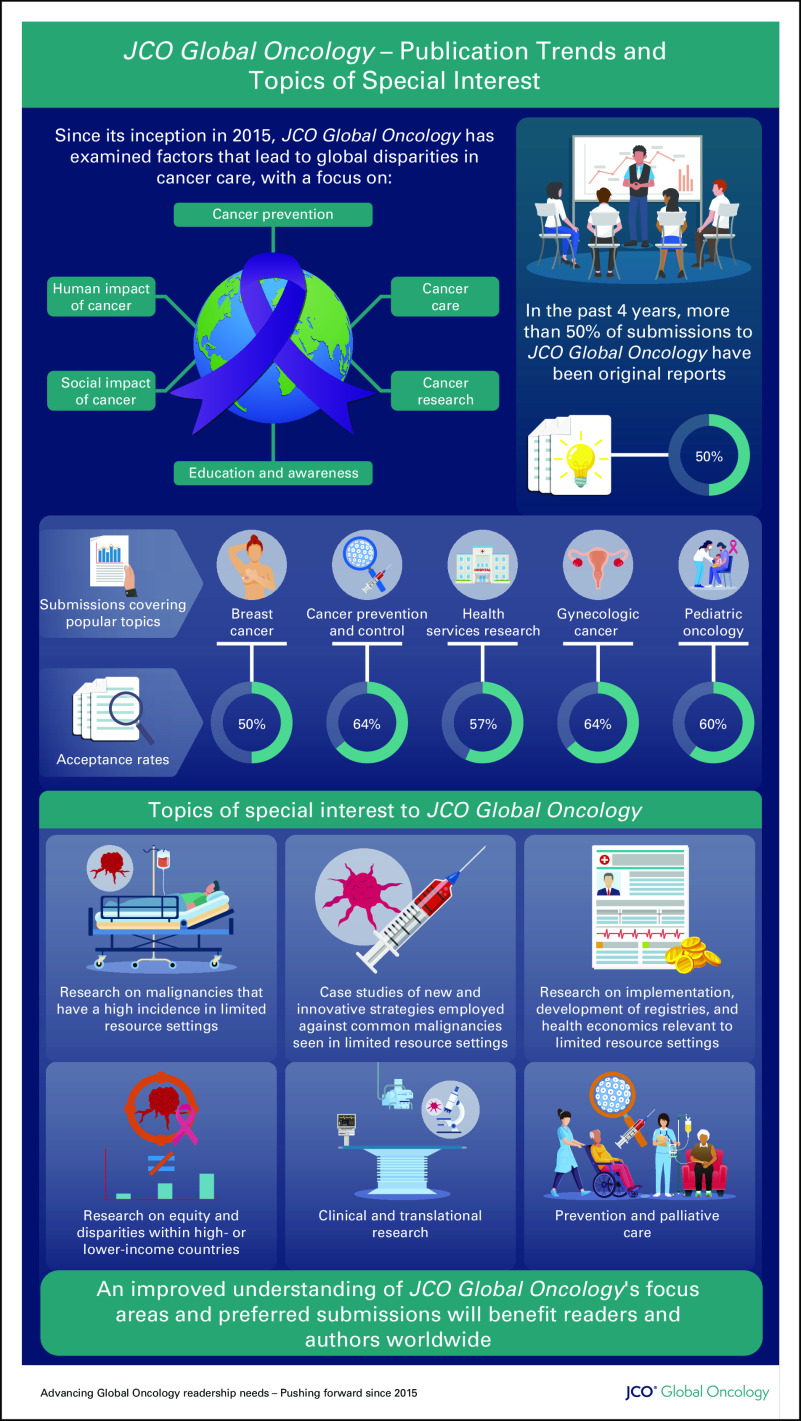
Publication trends and topics of interest to *JCO Global Oncology*.

Global oncology collaboratively addresses disparities and differences in cancer prevention, care, research, education, and the disease's social and human impact around the world encompassing a full spectrum of activities ranging from epidemiology to implementation of science to public health policy.^1^ *JCO Global Oncology (JGO)* since its inception in 2015 has published manuscripts highlighting these aspects aiming to contribute to reduce disparities in cancer care across the globe. In this editorial, we aim to provide clarity on preferred type of manuscripts that researchers in the global oncology might share to support our common goals. We built this on the early analysis of the trends and types of submissions in the first 2 operational years of *JGO* (Fig 1).

We, therefore, want to put forward a call for more submissions that focus on implementation science, biologic aspects of cancer in non-North American and Western European communities, development of cancer registries and data collections in settings with limited-resources, health economics, and policy research geared toward low- and middle-resource settings.^2^

We hope to achieve this goal by working with ASCO, the Union for International Cancer Control, and other organizations, our editorial boards, and stakeholders at large to seek more equitable representation. As an example, *JGO* quickly became the official outlet for ASCO's resource-stratified guidelines, authoritative manuscripts which respectfully guide regions, countries, and health care systems to ways and means to improve cancer control.

In continuing our mission, this editorial reviews the trends of successful submissions, 7 years after *JGO* inception to guide the next steps regarding preferable submissions for *JGO* publication for the benefit of our readers and authors who share our global oncology concerns and priorities.

## *JGO* Publication Trends

Original reports have been the most common type of submission in *JGO* with a steady increase to date consistently constituting more than 50% of manuscripts published in the past 4 years. Of note, there was a two-fold increase in 2020 compared with 2019, coinciding with the peak of the COVID-19 pandemic. The sudden halting of clinical activities during the pandemic and research personnel dedicating their time to academic writing probably were the main causes of this trend. Although the subsequent year, 2021 showed a dip in number of submissions coinciding with resumption of clinical duties, this was the year the most downloaded article, a record 202,414 times, in *JGO* was first published online. This was a paper describing the population-based registry from India, “Cancer Statistics, 2020: Report from National Cancer Registry Programme, India”^3^ representing an almost 10-fold over the previously most downloaded article (downloaded 23,092 times), the “ASCO resource–stratified guideline on primary prevention of cervical cancer.”^4^ Both articles align with the mission of *JGO* which continues to publish manuscripts focusing on population-based registries and ASCO's Global Oncology–oriented projects such as the resource stratified guidelines.

What other type of publications has *JGO* published in the past 3 years? Is the work being produced by academia in keeping with the values and interests of global oncology as guided by citations, downloads, and prior recommendations made 5 years ago? The top five submissions since 2019 were manuscripts on breast cancer (654), cancer prevention and control (552), health services research (371), gynecologic cancer (270), and pediatric oncology (265). The rates of acceptance of these top five submissions categories were 50%, 64%, 57%, 64%, and 60%, respectively. Although radiation oncology submissions were relatively common with a total of 178 manuscripts, this was one of the categories with the greatest acceptance rates at 82%. This possibly highlights a great demand for radiation oncology practice information, which readership may relate to from a resource capacity distribution perspective as per global oncology interest in a field highly driven by technology which threatens to widen disparities in quality of care if not supported equitably.

A few special series publications have been published. The largest one was on “Cancer Care in Indigenous Populations” with a total of 48 manuscripts and the smallest, with seven publications, was on genitourinary malignancies in low- and middle-income countries. Clinical trials are crucial in advancing evidence-based care and conversations of support of clinical trials with global inclusivity have been central in the oncology community, global oncology included. Forty submissions on clinical trials were submitted, and 28 (70%) were accepted. Cancer care costs are an important aspect of global oncology, and this is reflected by the relatively high rates of acceptance of manuscripts which address costs. Notably, 14 manuscripts on cost effectiveness were submitted, and 13 were accepted, and only 49 of the 63 submissions on economic analysis were accepted. However, given the volume of submissions received and the interests of our audience, case reports and single-center studies have not been prioritized.

These findings may be taken as a guide to potential authors on the types of papers the journal wishes to publish. Original reports will continue to be prioritized, but submissions of review articles, commentaries, correspondence and replies, special articles, and editorials are also welcomed. Topics of interest are listed in Table 1.

**TABLE 1:**
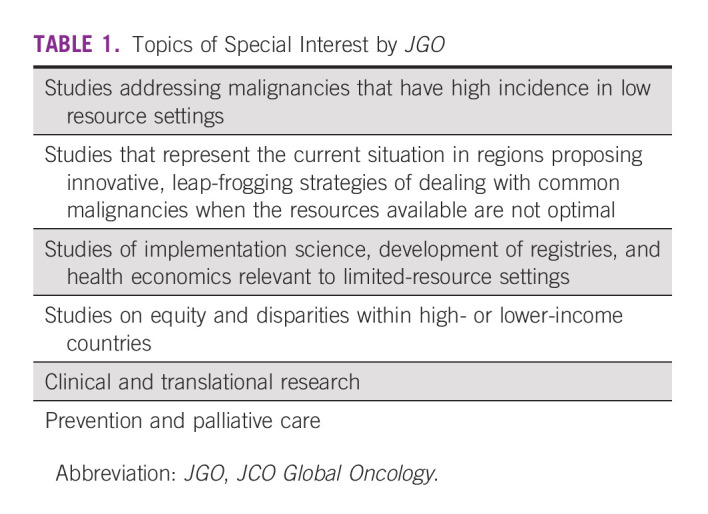
Topics of Special Interest by *JGO*

We end by extending our heartfelt thanks to our readers, authors, and reviewers for all the support they give *JGO* and hope this editorial will improve understanding of our focus and provide guidance on which papers are of interest to propel the values of global oncology. We welcome your comments and suggestions.
